# Assessing community pharmacists’ responses to pregnancy-related nausea and vomiting: A national simulated patient study in Jordan

**DOI:** 10.1371/journal.pone.0339327

**Published:** 2025-12-23

**Authors:** Khalid Al Kubaisi, Derar H. Abdel-Qader, Nadia Al Mazrouei, Abduelmula R. Abduelkarem, Yahya H. Dallal Bashi Dallal Bashi, Moh’d Ahmad Shara

**Affiliations:** 1 Department of Pharmacy Practice and Pharmacotherapeutics, College of Pharmacy, University of Sharjah, Sharjah, United Arab Emirates; 2 University of Petra, Amman, Jordan; The University of Lahore, PAKISTAN

## Abstract

**Background and aim:**

Nausea and vomiting of pregnancy (NVP) is the most common medical condition of gestation, affecting up to 90% of women and significantly impacting their quality of life. Community pharmacists (CPs) are often the first point of contact for these women, yet there is a lack of objective data on their practice quality in Jordan. This study aimed to conduct the first national, simulated patient study to assess objectively the assessment, management, counseling, satisfaction and predictors of appropriate practice among Jordanian community CPs when managing NVP.

**Materials and methods:**

A national, cross-sectional study using a simulated patient methodology was conducted in 380 community pharmacies, selected via proportionate stratified random sampling. Two validated scenarios (mild NVP and severe NVP with red flags) were used. A validated structured data collection form documented CPs ‘ assessment, management, counseling, and patient satisfaction. Multivariable logistic regression was used to identify independent predictors of “Appropriate Practice.” All data were analysed using SPSS (V28.0).

**Results:**

A significant gap between guideline-recommended care and actual practice was evident, particularly in high-risk situations. While most CPs (84.2%) initiated symptom inquiry, crucial assessment of red flags in the severe NVP scenario was dangerously low (e.g., inquiry about dehydration, 21.1%). This assessment failure translated directly to practice: only 56.8% of CPs correctly referred the high-risk patient, while 43.2% inappropriately sold an over-the-counter medication, delaying necessary medical care. Counseling on medication safety was consistently poor, with only 29.9% of CPs discussing potential side effects. Despite these clinical deficiencies, the overall patient satisfaction was high, appearing to be driven more by interpersonal skills than clinical accuracy. Multivariable analysis revealed that appropriate practice was independently predicted by prior maternal health training (aOR = 3.48, *p* < 0.001) and being a female pharmacist (aOR = 2.01, *p* = 0.009). Conversely, a high pharmacy workload was a significant independent barrier, reducing the odds of providing appropriate care by 50% (aOR = 0.50, *p* = 0.018).

**Conclusion:**

Jordanian community CPs are a critical but currently underperforming resource in maternal care. The prevalent gaps in clinical assessment and referral for severe NVP represent a significant patient safety risk. A one-size-fits-all approach to quality improvement is unlikely to succeed. Instead, a dual-pronged strategy is essential: (1) national professional pharmacy bodies must mandate targeted continuing professional development in maternal health, focusing on risk assessment and referral protocols; and (2) health policymakers and community pharmacy owners must address systemic barriers, particularly the detrimental impact of high workload on the delivery of safe and effective patient care.

## Introduction

Nausea and vomiting of pregnancy (NVP) is the most prevalent medical condition of gestation, affecting up to 90% of pregnant women worldwide [1]. While often self-limiting, the condition has a significant negative impact on women’s health-related quality of life, daily functioning, and work productivity [2,3]. In its most severe form, hyperemesis gravidarum (HG), NVP can lead to dehydration, electrolyte imbalances, and is a leading cause of hospitalisation in early pregnancy [4,5]. The profound psychosocial burden of NVP is also well-documented, with severe symptoms being associated with a reluctance to become pregnant again and, in some cases, consideration of pregnancy termination [2,6].

Community pharmacists (CPs) are particularly well-placed to provide first-contact care as they are easily accessible and are often consulted by women in early pregnancy, even before their first planned antenatal visit [7]. CPs play an important role in providing evidence-based advice regarding both OTC medications and non-pharmacological approaches, thus indicating they have the ability to safely and effectively manage NVP symptoms [8]. However, there is a growing body of evidence from cross-sectional studies published from many different countries, and international research, demonstrating that there are often significant gaps between evidence-based guidance and the care delivered within community pharmacy. There have been systematic reviews of SP studies which have shown gaps in the CPs assessment and counseling of patients and in the referral path for a variety of conditions [9,10].

This trend of sub-standard performance is also evident in the Middle East, where studies show that while pregnant women frequently consult CPs, the care provided is often suboptimal. For example, a study in Kuwait demonstrated that while CPs can provide services for NVP, some of their recommendations can be considered inappropriate in terms of unneeded drug therapy, or safety [11]. For instance, separate simulated patient (SP) studies in the United Arab Emirates also revealed significant omissions by CPs in the assessment and management of other conditions during pregnancy, such as migraine and asthma [12,13]. Although survey studies in the region suggest that CPs’ CPs believe they are well positioned in the pregnancy care continuum and are competent [14], objective data from SP studies invariably show a “perception-versus-reality” discrepancy that confirms “significant and remediable performance deficits” that can’t be identified by self-reported methods [15].

Despite this growing body of regional evidence, there remains a distinct lack of objective, practice-based data on the quality of NVP management by CPs CPs in Jordan. It is unknown how Jordanian CPs CPs assess women presenting with NVP, what treatments they recommend, and whether their practice aligns with current clinical guidelines. Therefore, this study aimed to fill this critical research gap by using SP methodology to objectively evaluate the current state of practice. The primary objectives are to: (1) assess the information-gathering skills of CPs; (2) evaluate the appropriateness of their management and treatment recommendations; (3) examine their counseling and communication practices; and (4) measure the level of patient satisfaction with the care provided for NVP.

## Materials and methods

### Study design and setting

This was a cross-sectional, observational study employing SP methodology. This design was selected as the gold standard for objectively assessing the actual practice behaviors of CPs in a naturalistic setting, thereby minimizing the potential for observation bias and social desirability bias that are inherent in self-reported surveys or overt observational methods. The study was conducted in community pharmacies that were licensed in the Hashemite Kingdom of Jordan. The data collection phase of the study was completed in the three-month period from June to August 2025.

### Study population, sampling strategy, and sample size

The target population of this study included all licensed CPs who worked in patient-facing roles in community pharmacies across the Hashemite Kingdom of Jordan. The sampling frame was the official registry of the Jordan Pharmacists Association. At the time of the study, it indicated there were approximately 32,727 licensed CPs in Jordan [16]. Those working in non-patient-facing roles (e.g., administrative, academic, or industry roles) and pharmacy students or interns were excluded.

In addition to determining the sample features (e.g., estimated target population, geographic consideration), we needed to determine a sample size that would be statistically representative of the target population. Using the Raosoft online sample size calculator (Raosoft, Inc., Seattle, WA, USA), we determined our minimum required sample size was 380 based on a 5% margin of error, a 95% confidence interval, and a conservative 50% response distribution to achieve the greatest variance in the sample.

A proportionate stratified random sampling method was used to identify the pharmacies included in the simulated patient visits. First, the sampling frame of all licensed community pharmacies was stratified into Jordan’s three administrative regions: North, Central, and South. We then used a computer-generated random number sequence, created using the research randomizer function in the Statistical Package for the Social Sciences (SPSS), to randomly select a pharmacy from each region. The number of pharmacies selected from each stratum was based on how many licensed community pharmacies were on the total list to maintain geographic representativeness. The participant in the study was the licensed pharmacist on duty when the simulated patient visited. If two or more pharmacists were on duty, the simulated patient was asked to approach the pharmacist who appeared most readily available to provide patient care.

### Data collection instrument and validation

Data collection was undertaken using a structured, concealed data collection tool, which was designed using a detailed literature review of both international and national clinical guidelines about managing NVP. The tool designed was informed by pharmacy consultation models established for pharmacy practice, including WWHAM [17] although this was amended to include clinical red flags specific for severe NVP.

WWHAM has been defined as: a well-known acronym/writing aid to help the pharmacist consider the patient’s choice when managing requests for over-the-counter medicines for managing minor ailments, and explicitly encourages the pharmacist to ask; **W**ho is this patient? **W**hat are the symptoms? **H**ow long have the symptoms been there?, **A**ction already taken?, **M**edication currently taking? For this research purpose only, WWHAM was amended to include clinical red flags specific for severe NVP.

The tool was intended to be comprehensive, and divided into four sections as follows:

- **Section A: Pharmacy and Pharmacist Characteristics:** Collected observable data on the pharmacist’s gender, years of experience, highest education and training courses, as well as pharmacy characteristics, such as type (independent vs. chain), location (urban vs. rural), and an estimate of workload at the time of the visit.
- **Section B: Assessment Checklist:** Assessed the pharmacist’s information-gathering process using a checklist of key questions regarding the patient’s symptoms (duration, timing, triggers), pregnancy status (trimester), and medical/medication history (other medications, allergies, previous treatments).
- **Section C: Management and Counseling:** Captured all management actions and recommendations, including whether a medication was dispensed, the appropriateness of the product choice, dose, and frequency. It also documented the provision of non-pharmacologic advice and specific safety-related counseling points (e.g., side effects, precautions/contraindications, and when to see a doctor).
- **Section D: Simulated Patient Satisfaction:** Utilized a series of six questions rated on a 5-point Likert-type scale (1 = *Very little* to 5 = *Very large*) to measure the SP’s perception of the consultation’s overall quality, usefulness, and the specific insights gained.

The data collection form and its accompanying scenarios underwent a rigorous validation process. Content validity was established by a multidisciplinary panel of four experts, including a senior clinical pharmacist, a senior practicing obstetrician, and two senior academic CPs with expertise in SP methodology, to ensure clinical accuracy and relevance to Jordanian practice.

A pilot study was then conducted, involving 10 SP visits to pharmacies not included in the final analysis. The pilot test aimed to: (1) assess the clarity and real-world feasibility of the scenarios, (2) ensure the data collection form was comprehensive and could be completed accurately and efficiently by the SPs, and (3) refine the SP training protocol. Minor modifications were made to the form’s layout and question wording based on pilot feedback to improve data recording efficiency.

Reliability of the data collection was ensured through the rigorous standardisation of the SPs’ performance via the intensive training programme. The use of a highly structured, checklist-based form minimised subjective interpretation and ensured consistent data capture across all visits.

### Simulated patient scenarios

Two standardized SP scenarios were designed following a thorough review of current international clinical guidelines on NVP management. These scenarios were then verified by a panel of a senior clinical pharmacist and a senior practicing obstetrician for clinical authenticity and pertinence to Jordanian community practice.

- Scenario 1: Mild NVP. The SP represented a woman in her 8th week of pregnancy with symptoms of persistent daily nausea but no vomiting for two weeks. She had not previously tried any treatments and asked for an over-the-counter (OTC) remedy. The expected optimal outcome was evidence-based non-pharmacological advice and recommendation for safe OTC product such as pyridoxine (Vitamin B6).
- Scenario 2: Severe NVP with Red Flags. The SP represented a woman in her 10th week of pregnancy with frequent vomiting (4–5 times/day) and unable to keep even fluids down as well as feelings of dizziness. The symptoms presented potentially indicated dehydration and a more severe condition or diagnosis, such as hyperemesis gravidarum, meaning they had speculation of potential red flags. The expected optimal outcome was direct and immediate referral to physician or emergency service without an OTC product sale.

### Simulated patient recruitment and training

Two pregnant female research assistants with health sciences backgrounds, who were not CPs and were unknown to the pharmacy community, were recruited to act as SPs. Both SPs underwent a comprehensive one-day (8-hour) training workshop to ensure consistency and reliability in scenario presentation. The training protocol was designed to achieve robust standardization of the SPs’ performance and included:

1. In-depth review of both NVP scenarios, including scripted histories and responses to anticipated questions.
2. Extensive role-playing exercises to standardize emotional affect, presentation of symptoms, and interaction style.
3. Instruction on probing techniques to be used only if the pharmacist failed to ask critical questions.
4. Protocols for handling situations where the pharmacist might detect the simulation, to ensure a polite and neutral exit.
5. Thorough training on the immediate and accurate completion of the electronic data collection form to minimize recall bias.

The pilot study, which involved 10 pharmacy visits, served as a final validation step to confirm the real-world feasibility of the scenarios and to validate the SPs’ training and adherence to the protocol before the main data collection phase began.

### Data collection procedure and post-hoc consent

The data collection was conducted over a 12-week period, from June to August 2025. The two trained SPs were randomly assigned to the selected pharmacies within each geographic stratum to ensure balanced data collection.

The study employed a two-step data collection procedure for each pharmacy visit to ethically link observed practice with pharmacist characteristics while maintaining the integrity of the initial covert assessment.

**Step 1: Covert SP Interaction**: The SP entered the pharmacy and enacted their assigned scenario covertly. During this interaction, the SP’s primary role was to perform the scenario consistently and identify the participating pharmacist. Immediately after exiting the pharmacy, the SP recorded the pharmacist’s clinical performance and counseling actions on the structured electronic data collection form. This performance data was assigned a unique visit code. To measure the consultation duration, the SP discreetly used a stopwatch function on a mobile phone, starting upon initiating the conversation with the pharmacist and stopping upon exiting. This duration was immediately recorded in the data collection form.

**Step 2: Immediate Revisit, Debriefing, and Verbal Consent**: Within 5 minutes of the initial interaction, the same SP re-entered the pharmacy and approached the pharmacist who had served them. The SP then initiated a standardized debriefing protocol:

1. The SP re-introduced themselves as a research assistant for a national study on pharmacy practice.
2. The SP then disclosed the nature of the visit and asked for permission to include the anonymous data from the interaction in the research.
3. Crucially, the SP then requested verbal informed consent. If the pharmacist provided verbal consent, the SP then asked for the demographic information.

The SP recorded the consent status and the demographic data on the same electronic form, linking it directly to the performance data via the unique visit code. This procedure, including the waiver of prior written consent and the method for documenting post-encounter verbal consent, was explicitly reviewed and approved by the University of Petra’s Institutional Review Board (IRB). If consent was denied, the SP thanked the pharmacist for their time and immediately marked the entire record for deletion from the final dataset. Data from 40 encounters were excluded because the pharmacist declined to participate in the study.

### Outcome measures and variables

- **Primary Outcome:** The primary outcome was appropriate practice, a binary variable defined based on the scenario.
  - For Scenario 1 (Mild NVP), it was defined as recommending an evidence-based OTC treatment (pyridoxine +/- antihistamine) *and* providing at least two distinct points of non-pharmacological advice.
  - For Scenario 2 (Severe NVP), it was defined as a direct referral to a physician or emergency service without the sale of any OTC medication.
- **Secondary Outcomes:** These included the frequency of key assessment questions, the types of medications recommended, the provision of specific counseling points, and the SP’s satisfaction score.
- **Independent Variables:** Predictor variables included pharmacist’s gender, years of experience, highest educational degree, prior training in maternal health, pharmacy type (independent vs. chain), and pharmacy location (north, central, south).

### Ethical considerations

The study protocol received full approval from the Institutional Review Board (IRB) at the University of Petra (S/9/6/2025). Given that overt observation would fundamentally alter pharmacist behaviour and invalidate the results, a waiver of prior informed consent was granted by the IRB, a standard practice for this methodology. To protect confidentiality, no names of CPs or pharmacies were recorded. All data were anonymised and aggregated before analysis, ensuring that no individual or establishment could be identified in the study’s reporting.

### Statistical analysis

All collected data were coded, cleaned, and analysed using the SPSS, Version 28.0 (IBM Corp., Armonk, NY).

Descriptive statistics (frequencies, percentages, medians, and interquartile ranges (IQR)) were used to summarize all demographic and outcome variables. Inferential statistics were used to assess associations between pharmacist groups and the main outcomes. Specifically, the Chi-square test (or Fisher’s exact test) was employed for comparing frequencies of categorical variables between the two scenarios. The Mann-Whitney U test was used for non-normally distributed continuous variables, such as the patient satisfaction scores. The normality of distribution for continuous variables, such as patient satisfaction scores, was assessed using the Shapiro-Wilk test. As the data were found to be non-normally distributed (p < 0.05), non-parametric tests were employed for inferential analysis.

To identify independent predictors of the primary outcome, Appropriate Practice, a multivariable logistic regression model was constructed. A two-step process was employed: first, univariate logistic regression was performed for each potential predictor. Variables with a p-value < 0.1 in the univariate analysis were considered for inclusion in the final multivariable model [18]. An assessment for multicollinearity was conducted on the variables selected for the model; all Variance Inflation Factors (VIFs) were < 5, indicating that no significant collinearity was present. The results of the regression analysis are reported as both Crude Odds Ratios (COR) and Adjusted Odds Ratios (aOR) with their corresponding 95% confidence intervals (CIs). For all final inferential tests, a two-tailed p-value of ≤ 0.05 was considered statistically significant.

## Results

A total of 380 community CPs successfully participated in the simulated patient study, achieving the calculated required sample size. The visits were evenly distributed between the two standardised scenarios: a mild NVP case (Scenario 1, n = 190) and a severe NVP case featuring red flags that necessitated physician referral (Scenario 2, n = 190).

### Pharmacist and pharmacy characteristics

A total of 380 community pharmacists, from an initial 420 SP visits, provided verbal informed consent during an immediate post-encounter debriefing and were included in the final analysis.

**Fig 1:**
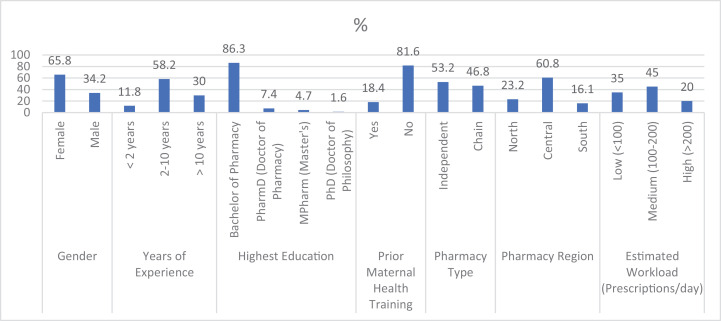
Demographic and characteristics of participating pharmacists (N = 380).

The demographic and professional characteristics of the participating CPs CPs are detailed in Fig 1. The sample was predominantly female (65.8%), with a median of 8 years of professional experience. A substantial majority (81.6%) had not received any formal postgraduate training specifically in maternal or prenatal healthcare. The study included a representative mix of pharmacy types, with 53.2% being independent and 46.8% part of a chain, primarily located in Jordan’s densely populated central region.

### Pharmacist assessment practices

The information-gathering and assessment skills of CPs were evaluated based on a predefined set of essential questions related to NVP. The performance was inconsistent, with basic symptom inquiry being common but a comprehensive clinical history frequently being omitted.

The most consistently asked questions were related to the patient’s pregnancy status and trimester, which was confirmed in 76.6% of encounters, and the patient’s age (72.1%). Questions about the core characteristics of the symptoms, such as their duration and timing, were asked in just over half of the interactions (Table 1).

**Table 1:** Frequency of assessment questions asked by community pharmacists during SP encounters.

Assessment Criteria	Scenario 1 (N = 190) n (%)	Scenario 2 (N = 190) n (%)	Overall (N = 380) n (%)	*p*-value
**Pregnancy & Patient Details**				
Confirmed pregnancy status and trimester	150 (78.9)	141 (74.2)	291 (76.6)	0.315
Asked about the age of the SP	141 (74.2)	133 (70.0)	274 (72.1)	0.401
**Symptom Assessment**				
Asked about the timing of nausea/vomiting	119 (62.6)	96 (50.5)	215 (56.6)	0.045*
Asked about the duration of the symptoms	125 (65.8)	102 (53.7)	227 (59.7)	0.023*
Asked about triggers of NVP	52 (27.4)	46 (24.2)	98 (25.8)	0.528
Asked about associated symptoms (e.g., pain, fever)	50 (26.3)	58 (30.5)	108 (28.4)	0.399
**Medical & Treatment History**				
Asked about other medications taken	90 (47.4)	75 (39.5)	165 (43.4)	0.144
Asked if anything was already tried for the symptoms	81 (42.6)	60 (31.6)	141 (37.1)	0.048*
Asked about patient’s allergy reactions	68 (35.8)	60 (31.6)	128 (33.7)	0.431

*Chi-square test was used to determine statistical significance between scenarios; p ≤ 0.05 is considered statistically significant.

Significant gaps were observed in the collection of a complete medical and medication history. Less than half of the CPs (43.4%) asked about other medications the patient might be taking, and only about one-third inquired about previous treatments tried (37.1%) or asked about patient allergies (33.7%). Questions regarding potential triggers for NVP or associated symptoms like fever or pain were the least frequently asked, occurring in less than 30% of visits.

Statistically significant differences were noted between the two scenarios. Pharmacists were more likely to inquire about the duration (p = 0.023) and timing (p = 0.045) of symptoms in the mild NVP scenario compared to the severe NVP scenario. Conversely, inquiry about associated symptoms was slightly higher in the severe case, though this difference was not statistically significant. This pattern of limited information gathering directly influenced the subsequent management and counseling provided to the simulated patients.

### Management actions, non-pharmacological advice, and referral

Pharmacists’ primary management actions varied significantly based on the clinical scenario presented. Out of 380 total encounters, a medication was sold in 66.8% of cases, while a referral to a doctor was made in 30.5% of cases. The appropriateness of these actions was highly dependent on the scenario.

As shown in Table 2, in the mild NVP scenario, CPs appropriately chose to provide treatment, dispensing a medication in 90.5% of visits. In contrast, for the severe NVP scenario, the correct action of referring the patient to a physician was only performed in 56.8% of encounters. Concerningly, in the remaining 43.2% of these high-risk cases, CPs sold a medication instead of referring, thereby delaying necessary medical evaluation.

**Table 2:** Management actions and non-pharmacological advice by scenario (N = 380).

Management Action	Scenario 1 (N = 190) n(%)	Scenario 2 (N = 190) n (%)	Overall (N = 380) n (%)	*p*-value
Pharmacist Dispensed/Sold a Medicine	172 (90.5)	82 (43.2)	254 (66.8)	<0.001*
Pharmacist Referred to the Doctor	8 (4.2)	108 (56.8)	116 (30.5)	<0.001*
Advised Non-Pharmacologic Therapy (e.g., dietary changes, food advice)	116 (61.1)	35 (18.4)	151 (39.7)	<0.001*

*Chi-square test was used to determine statistical significance between scenarios; p ≤ 0.05 is considered statistically significant.

Non-pharmacologic therapy, a key component of NVP care, was advised significantly more often in the mild scenario (61.1%) than in the severe scenario (18.4%), highlighting a missed opportunity for holistic patient education.

### Quality of counseling for dispensed medications

This section analyses the 254 encounters where a CP dispensed a medication (172 visits from Scenario 1 and 82 from Scenario 2).

As detailed in Table 3, while the technical aspects of the recommendations were generally strong, counseling on crucial safety information was severely lacking. The appropriateness of the selected product, its dose, and frequency all adhered to therapeutic guidelines in over 84% of cases.

**Table 3:** Quality of Counseling in Encounters Where a Medication Was Dispensed (N = 254).

Counseling Point & Quality Metric	Scenario 1 (N = 172) n(%)	Scenario 2 (N = 82) n (%)	Overall (N = 254) n (%)	*p*-value
Adherence to Therapeutic Guidelines (Product Choice)	155 (90.1)	66 (80.5)	221 (87.0)	0.031*
Dose Was Appropriate	165 (95.9)	77 (93.9)	242 (95.3)	0.485
Frequency Was Appropriate	147 (85.5)	68 (82.9)	215 (84.6)	0.611
Told about Adverse Drug Reactions or Side Effects	52 (30.2)	24 (29.3)	76 (29.9)	0.881
Told about Precautions/ Contraindications	27 (15.7)	12 (14.6)	39 (15.4)	0.834
Average Dispensing Time (Median, IQR)	105s (75–140s)	80s (55–110s)	95s (65–130s)	0.012*

*p-value ≤ 0.05 is considered statistically significant (Chi-square test for categorical data; Mann-Whitney U test for dispensing time).

However, communication about patient safety was consistently poor across both scenarios. Overall, only 29.9% of CPs discussed potential adverse drug reactions or side effects. Even fewer, just 15.4%, mentioned necessary precautions or contraindications for the dispensed medicine. There were no statistically significant differences between the mild and severe scenarios in the provision of this vital safety information, indicating that the presence of higher-risk symptoms did not prompt more thorough safety counseling. The median time for consultations involving a sale was brief, at just 95 seconds.

### Simulated patient satisfaction with the pharmacist’s consultation

Despite the observed gaps in clinical assessment and guideline adherence, the SPs satisfaction with the overall quality of the CP consultations was generally positive. The findings suggested that interpersonal skills and perceived helpfulness were strong drivers of patient satisfaction, even when clinical management was suboptimal.

As detailed in Table 4, when asked about their overall satisfaction with the consultation, 73.7% of encounters were rated as “large” or “very large.” Similarly, 72.4% of SPs found the consultation “useful” to a large or very large extent.

**Table 4:** Simulated Patient Satisfaction with Pharmacist Consultation by Scenario (N = 380).

Satisfaction Question	Very little n (%)	Little n (%)	Neutral n (%)	Large n (%)	Very large n (%)	Overall (Large + Very large) n (%)	*p*-value
**1. Overall Satisfaction**							0.002*
Scenario 1 (n = 190)	3 (1.6)	9 (4.7)	31 (16.3)	98 (51.6)	49 (25.8)	147 (77.4)	
Scenario 2 (n = 190)	11 (5.8)	22 (11.6)	48 (25.3)	76 (40.0)	33 (17.4)	109 (57.4)	
*Overall (N = 380)*	*14 (3.7)*	*31 (8.2)*	*79 (20.8)*	*174 (45.8)*	*82 (21.6)*	*256 (67.4)*	
**2. Consultation Usefulness**							<0.001*
Scenario 1 (n = 190)	5 (2.6)	11 (5.8)	35 (18.4)	102 (53.7)	37 (19.5)	139 (73.2)	
Scenario 2 (n = 190)	14 (7.4)	25 (13.2)	59 (31.1)	65 (34.2)	27 (14.2)	92 (48.4)	
*Overall (N = 380)*	*19 (5.0)*	*36 (9.5)*	*94 (24.7)*	*167 (43.9)*	*64 (16.8)*	*231 (60.8)*	
**3. Consultation Worth Time Spent**							0.015*
Scenario 1 (n = 190)	6 (3.2)	15 (7.9)	40 (21.1)	95 (50.0)	34 (17.9)	129 (67.9)	
Scenario 2 (n = 190)	12 (6.3)	28 (14.7)	55 (28.9)	71 (37.4)	24 (12.6)	95 (50.0)	
*Overall (N = 380)*	*18 (4.7)*	*43 (11.3)*	*95 (25.0)*	*166 (43.7)*	*58 (15.3)*	*224 (58.9)*	
**4. Insight into How to Use Medications**							<0.001*
Scenario 1 (n = 190)	10 (5.3)	28 (14.7)	55 (28.9)	75 (39.5)	22 (11.6)	97 (51.1)	
Scenario 2 (n = 190)	31 (16.3)	45 (23.7)	71 (37.4)	35 (18.4)	8 (4.2)	43 (22.6)	
*Overall (N = 380)*	*41 (10.8)*	*73 (19.2)*	*126 (33.2)*	*110 (28.9)*	*30 (7.9)*	*140 (36.8)*	
**5. Found a Solution to Problems/Concerns**							<0.001*
Scenario 1 (n = 190)	7 (3.7)	15 (7.9)	33 (17.4)	99 (52.1)	36 (18.9)	135 (71.1)	
Scenario 2 (n = 190)	25 (13.2)	48 (25.3)	44 (23.2)	51 (26.8)	22 (11.6)	73 (38.4)	
*Overall (N = 380)*	*32 (8.4)*	*63 (16.6)*	*77 (20.3)*	*150 (39.5)*	*58 (15.3)*	*208 (54.7)*	
**6. Insight into Managing/Treating NVP**							<0.001*
Scenario 1 (n = 190)	9 (4.7)	20 (10.5)	49 (25.8)	88 (46.3)	24 (12.6)	112 (58.9)	
Scenario 2 (n = 190)	22 (11.6)	39 (20.5)	65 (34.2)	51 (26.8)	13 (6.8)	64 (33.7)	
*Overall (N = 380)*	*31 (8.2)*	*59 (15.5)*	*114 (30.0)*	*139 (36.6)*	*37 (9.7)*	*176 (46.3)*	

*p-value calculated using the Mann-Whitney U test to compare the distribution of satisfaction ratings between Scenario 1 and Scenario 2. p ≤ 0.05 is considered statistically significant.

However, satisfaction levels were notably lower for questions related to gaining specific, actionable insights. For instance, only 47.9% of SPs felt the consultation gave them a “large” or “very large” degree of insight into how to use medications during pregnancy. Similarly, just 51.8% felt they gained significant insight into how to manage their NVP symptoms.

A statistically significant difference in satisfaction was observed between the two clinical scenarios. This comparison was intentionally designed to explore whether patient satisfaction was driven more by receiving a tangible product or by receiving a clinically appropriate recommendation (i.e., referral). Overall satisfaction was significantly higher in the mild NVP scenario (Scenario 1) compared to the severe NVP scenario (Median score 4 vs. 4, p = 0.002). This difference was most pronounced for the question, “To what extent did you find a solution to your problems/concerns?”, where 71.1% of SPs in Scenario 1 reported a “large” or “very large” extent, compared to only 38.3% in Scenario 2 (p < 0.001). This suggested that receiving a tangible product in the mild NVP case was perceived as more satisfying than receiving a referral for the severe case.

### Predictors of appropriate practice

To identify factors associated with “appropriate practice,” both univariate and multivariable logistic regression analyses were performed. The primary outcome was a binary variable indicating whether the CP correctly managed the NVP scenario. Variables with a *p*-value < 0.1 in the univariate analysis were included in the final multivariable model to adjust for potential confounding effects.

The results of both the crude and adjusted analyses are presented in Table 5. In the univariate (crude) analysis, five factors were significantly associated with a higher likelihood of providing appropriate care: being a female pharmacist (Crude Odds Ratio (COR) = 2.01), having prior maternal health training (COR = 3.78), practicing in a chain pharmacy (COR = 1.55), having more years of experience, and having a lower pharmacy workload. Pharmacist’s highest education level and the geographic region of the pharmacy were not significantly associated with the outcome and were therefore excluded from the multivariable analysis.

**Table 5:** Univariate and multivariable logistic regression analysis of predictors for appropriate practice (N = 380).

Characteristic	Crude Odds Ratio (COR) (95% CI)	*p*-value	Adjusted Odds Ratio (aOR) (95% CI)	*p*-value
**Gender**				
Male	1.00 (Ref)	–	1.00 (Ref)	–
Female	2.01 (1.20–3.37)	0.015*	2.01 (1.19–3.41)	0.009**
**Years of Experience**				
< 2 years	1.00 (Ref)	0.091*	1.00 (Ref)	–
2-10 years	1.62 (0.91–2.89)		1.51 (0.84–2.72)	0.168
> 10 years	1.75 (0.95–3.23)		1.39 (0.74–2.61)	0.310
**Highest Education**				
Bachelor of Pharmacy	1.00 (Ref)	0.234	–	–
PharmD/ MSc/ PhD	1.38 (0.81–2.35)		–	–
**Prior Maternal Health Training**				
No	1.00 (Ref)	–	1.00 (Ref)	–
Yes	3.78 (2.29–6.24)	<0.001*	3.48 (2.08–5.84)	<0.001**
**Pharmacy Type**				
Independent	1.00 (Ref)	0.088*	1.00 (Ref)	–
Chain	1.55 (0.94–2.56)		1.46 (0.89–2.38)	0.131
**Pharmacy Region**				
Central	1.00 (Ref)	0.451	–	–
North	0.88 (0.54–1.44)		–	–
South	1.23 (0.70–2.16)		–	–
**Estimated Workload (Prescriptions/day)**				
Low (<100)	1.00 (Ref)	0.009*	1.00 (Ref)	–
Medium (100–200)	0.71 (0.48–1.02)	0.080*	0.75 (0.50–1.13)	0.150
High (>200)	0.45 (0.27–0.75)	0.005*	0.50 (0.29–0.86)	0.018*

*Statistically significant p-values (≤ 0.1) in the univariate analysis, identifying variables for inclusion in the multivariable model.

**Statistically significant p-values (≤ 0.05).

Note: An assessment for multicollinearity was conducted prior to the final analysis; all Variance Inflation Factors (VIFs) for the variables included in the multivariable model were < 5; indicating no significant collinearity.

After adjusting for the significant variables in the multivariable model, three factors remained as independent predictors of appropriate practice. The strongest predictor was prior maternal health training, with trained CPs being over three times more likely to provide appropriate care (aOR = 3.48, *p* < 0.001). Being a female pharmacist also remained a significant predictor (aOR = 2.01, *p* = 0.009). Finally, a high pharmacy workload was independently associated with a 50% reduction in the odds of providing appropriate care compared to a low workload setting (aOR = 0.50, *p* = 0.018).

## Discussion

The primary aim of this national SP study was to assess objectively the current practice of community pharmacists (CPs) in Jordan regarding the assessment, management, and counseling of women with NVP. The study revealed a significant gap between evidence-based guidelines and the actual care provided. Key findings included widespread deficiencies in patient assessment, particularly in identifying clinical red flags; a concerningly low rate of appropriate physician referral for severe NVP cases; and consistently inadequate safety counseling for dispensed medications.

The findings of this study, while specific to Jordan, were remarkably consistent with a growing body of international and regional research that highlights systemic challenges in community pharmacy practice [19,20]. The use of the SP methodology provides an objective lens, confirming practice deficiencies that have been previously self-reported or identified through other means [9,10,15].

The management of NVP in our study was suboptimal, particularly in high-risk cases. The failure of 43.2% of CPs to refer a patient with clear red flags for hyperemesis gravidarum (HG) is a critical patient safety concern, as untreated HG is a leading cause of hospitalisation in early pregnancy [4,6]. This finding is echoed in other regional SP studies. A study in Sudan on acute diarrhea found that referral for severe cases was “below expectation” [21], suggesting a broader difficulty in recognising the boundaries of self-care.

Even when medication was appropriately recommended for mild NVP, counseling was minimal. The lack of advice on side effects and precautions is a recurring theme. Studies in Kuwait and the UAE consistently show that while CPs are frequently consulted during pregnancy, their advice is often incomplete [7,11]. Furthermore, the lack of non-pharmacological advice in our study (provided in only 39.7% of encounters) is consistent with a comparative study between Serbia and Norway, where non-pharmacological counseling was also found to be insufficient [22]. The limited effectiveness of community pharmacy interventions noted in a systematic review by Blalock et al. [8] may be partly explained by this failure to implement comprehensive, guideline-based care that includes both pharmacological and lifestyle components.

Our regression analysis provided objective, quantitative evidence for barriers that have been consistently self-reported in qualitative and survey-based research. The finding that a high workload was an independent predictor of poor practice (aOR = 0.50) gives weight to the “lack of time” cited as a major barrier by CPs in studies from Australia, Belgium, and the UAE [23,24,14]. This indicates that any systemic pharmacy context issues can potentially overpower individual pharmacist capability.

The strongest predictor of appropriate practice was previous maternal health training (aOR = 3.48). This speaks to the most frequently identified barrier in regional surveys; lack of specific training and the knowledge of updated guidelines [14,25]. A study conducted in Palestine also found that being a participant in CPD programmes correlated with higher knowledge scores for drug safety in pregnancy [25]. Therefore, this presents a strong argument that targeted and ongoing education is not just beneficial, but essential in achieving improved maternal health services.

Finally, the phenomenon of high patient satisfaction despite poor clinical care, has been well-documented in SP research [10]. It suggests patients value interpersonal skills as much, if not more than, clinical accuracy. At the same time, this could have implications for a wider uncertainty of the CP’s clinical role. Leung et al. [23] noted that Australian CPs believe they play a secondary role to general practitioners; this may result in CPs not feeling able to perform comprehensive clinical assessments. Therefore, building a professional identity that includes clinical responsibility, is equally important to imparting technical knowledge.

Although there were a number of methodological strengths in this study that would improve the validity and relevancy of the findings, there was one significant methodological strength, being the SP (also known as standardized patient or clinical role play) methodology, which is considered the gold standard methodological approach for objectively assessing clinical practice. The SP methodology illustrates CPs real behaviors, in their own clinical work place, which reduces the socially desirable and recall biases that occurs in survey or self-reported based research. Also, the study had national reach, which allows for generalization of the study findings. The sampling strategy employed was sound; it used a proportionate stratified random sampling strategy from Jordan’s diverse geographic regions and range of practice settings as it achieved a sample that is represented the CP population and enables the study findings to more confidently generalize to provide national-level informed policy and educational reform. The study was also well-supported by inclusion of two clinical scenarios. The two scenarios allowed the study to assess a range of professional competencies, from basic routine counselling for a common condition, to key clinical judgement identifying and for keeping a high-risk client.

Despite its methodological strengths, particularly the use of a robust SP design to capture actual practice, this study has several limitations that should be acknowledged. First, while every effort was made to ensure the SP encounters were covert, the possibility of pharmacist detection cannot be entirely ruled out. Any such detection could have induced the Hawthorne effect, leading some CPs to perform better than their typical practice. Second, the findings are specific to the two NVP scenarios presented; CPs’ assessment and counseling behaviours may differ when faced with other pregnancy-related ailments or more complex clinical presentations. Furthermore, the data collection relied on the SPs’ immediate documentation using a structured form. While this method is reliable for capturing predefined actions, it does not capture the nuances of verbal and non-verbal communication that could be obtained through audio or video recordings. Finally, this study was designed to observe what CPs did, but it could not explain why they make certain decisions. Future research employing a mixed-methods approach, such as qualitative interviews with CPs following the SP visits, would be invaluable for exploring the underlying reasons for the practice gaps identified, whether they stem from knowledge deficits, attitudinal barriers, or systemic pressures like workload.

## Conclusion

In conclusion, this study reveals a critical disconnect between the potential and actual role of Jordanian community pharmacists in maternal care. Significant gaps in clinical assessment, failure to identify red flags for severe NVP, and inadequate safety counseling represent a tangible risk to patient safety. Notably, the study identified that prior maternal health training, female gender, and a lower workload were significant independent predictors of appropriate practice. This suboptimal practice delays necessary medical intervention and undermines the principles of safe medication use. These findings are a clear call to action for key stakeholders. Pharmacy education and professional bodies must implement mandatory continuing professional development (CPD) focused on enhancing clinical assessment and risk-recognition skills. Simultaneously, policymakers and pharmacy owners must address systemic barriers, particularly the detrimental impact of high workload, to create an environment that supports quality patient care. By investing in targeted training and supportive practice models, the Jordanian pharmacy profession can bridge these identified gaps and make a more significant contribution to the health and safety of pregnant women.

## Supporting information

S1 File Anonymized Dataset. (XLSX)

S2 File Inclusivity in Global Research. (DOCX)

S3 File Questionnaire. (DOCX)
