# Supplementary material for: Assessing community pharmacists’ responses to pregnancy-related nausea and vomiting: A national simulated patient study in Jordan
Source: PLoS One. 2025 Dec 23;20(12):e0339327. doi: 10.1371/journal.pone.0339327 (PMC12725735; doi:10.1371/journal.pone.0339327)
Supplement: S3 File — (DOCX) [file pone.0339327.s003.docx]

# Questionnaire

**Study Title:** Assessing Community Pharmacists' Responses to Pregnancy-Related Nausea and Vomiting: A National Simulated Patient Study in Jordan

**Administrative Details (To be completed by SP before and immediately after visit)**

- **Unique Visit Code:** _________________________
- **Date of Visit:** (DD/MM/YYYY) **/**/________
- **Time of Visit:** (HH:MM) **:**
- **Scenario Enacted:**

Scenario 1: Mild NVP

Scenario 2: Severe NVP with Red Flags

- **Total Consultation Time (seconds):** __________

### Section A: Pharmacy and Pharmacist Characteristics (Observable Data)

1. **Pharmacy Type:**

Independent

Chain

1. **Pharmacy Region:**

North

Central

South

1. **Participating Pharmacist's Gender:**

Male

Female

1. **Estimated Years of Professional Experience (based on post-interaction debriefing):**

< 2 years

2-10 years

> 10 years

1. **Highest Education (based on post-interaction debriefing):**

Bachelor of Pharmacy

PharmD

MSc

PhD

1. **Prior Maternal Health Training (based on post-interaction debriefing):**

Yes

No

1. **Estimated Pharmacy Workload at Time of Visit:**

Low (<100 prescriptions/day)

Medium (100-200 prescriptions/day)

High (>200 prescriptions/day)

### Section B: Assessment Checklist (Pharmacist's Information Gathering)

*Instructions: For each item below, check "Yes" if the pharmacist asked the question or "No" if they did not.*

| Assessment Question | Yes | No |
| --- | --- | --- |
| **Pregnancy & Patient Details** |  |  |
| 1. Asked to confirm pregnancy status and/or trimester | [ ] | [ ] |
| 2. Asked about your age | [ ] | [ ] |
| **Symptom Assessment** |  |  |
| 3. Asked about the timing of nausea/vomiting | [ ] | [ ] |
| 4. Asked about the duration of the symptoms | [ ] | [ ] |
| 5. Asked about potential triggers for the symptoms | [ ] | [ ] |
| 6. Asked about associated symptoms (e.g., pain, fever) | [ ] | [ ] |
| **Medical & Treatment History** |  |  |
| 7. Asked about other medications you are currently taking | [ ] | [ ] |
| 8. Asked if anything was already tried for the symptoms | [ ] | [ ] |
| 9. Asked about patient's allergies | [ ] | [ ] |

### Section C: Management Actions and Counseling

1. **Primary Management Action Taken by Pharmacist:** (Select one)

Dispensed/Sold an OTC medication

Referred to a doctor / emergency service

Provided non-pharmacologic therapy

**--- COMPLETE THE FOLLOWING SECTION ONLY IF A MEDICATION WAS DISPENSED/SOLD ---**

1. **Appropriateness of Recommendation:**
   - Adherence to Therapeutic Guidelines (Product Choice): [ ] Appropriate [ ] Inappropriate
   - Dose Recommended: [ ] Appropriate [ ] Inappropriate
   - Frequency Recommended: [ ] Appropriate [ ] Inappropriate
2. **Safety Counseling Provided:**
   - Did the pharmacist tell you about adverse drug reactions or side effects?

Yes

No

- - Did the pharmacist tell you about necessary precautions or contraindications?

Yes

No

### Section D: Simulated Patient Satisfaction

*Instructions: Please rate the following aspects of your consultation on a scale of 1 to 5, where:*

- **1 = Very Little**
- **2 = Little**
- **3 = Neutral**
- **4 = Large**
- **5 = Very Large**

| Satisfaction Question | 1 | 2 | 3 | 4 | 5 |
| --- | --- | --- | --- | --- | --- |
| 1. **Overall Satisfaction:** To what extent were you satisfied with the consultation? | [ ] | [ ] | [ ] | [ ] | [ ] |
| 2. **Consultation Usefulness:** To what extent was the consultation useful? | [ ] | [ ] | [ ] | [ ] | [ ] |
| 3. **Worth Time Spent:** To what extent was the consultation worth the time you spent? | [ ] | [ ] | [ ] | [ ] | [ ] |
| 4. **Insight on Medications:** To what extent did you gain insight into how to use medications? | [ ] | [ ] | [ ] | [ ] | [ ] |
| 5. **Found a Solution:** To what extent did you find a solution to your problems/concerns? | [ ] | [ ] | [ ] | [ ] | [ ] |
| 6. **Insight on Managing NVP:** To what extent did you gain insight into managing/treating your NVP? | [ ] | [ ] | [ ] | [ ] | [ ] |
